# Examining Response to Negative Life Events Through Fitness Tracker Data

**DOI:** 10.3389/fdgth.2021.659088

**Published:** 2021-05-21

**Authors:** Louis Faust, Keith Feldman, Suwen Lin, Stephen Mattingly, Sidney D'Mello, Nitesh V. Chawla

**Affiliations:** ^1^Department of Computer Science & Engineering, University of Notre Dame, Notre Dame, IN, United States; ^2^Children's Mercy Kansas City, Kansas City, MO, United States; ^3^Department of Pediatrics, University of Missouri-Kansas City School of Medicine, Kansas City, MO, United States; ^4^Institute of Cognitive Science, University of Colorado, Boulder, CO, United States

**Keywords:** fitness trackers, negative life events, physical health, coping, Garmin, wearable technology

## Abstract

Negative life events, such as the death of a loved one, are an unavoidable part of life. These events can be overwhelmingly stressful and may lead to the development of mental health disorders. To mitigate these adverse developments, prior literature has utilized measures of psychological responses to negative life events to better understand their effects on mental health. However, psychological changes represent only one aspect of an individual's potential response. We posit measuring additional dimensions of health, such as physical health, may also be beneficial, as physical health itself may be affected by negative life events and measuring its response could provide context to changes in mental health. Therefore, the primary aim of this work was to quantify how an individual's physical health changes in response to negative life events by testing for deviations in their physiological and behavioral state (PB-state). After capturing post-event, PB-state responses, our second aim sought to contextualize changes within known factors of psychological response to negative life events, namely coping strategies. To do so, we utilized a cohort of professionals across the United States monitored for 1 year and who experienced a negative life event while under observation. Garmin Vivosmart-3 devices provided a multidimensional representation of one's PB-state by collecting measures of resting heart rate, physical activity, and sleep. To test for deviations in PB-state following negative life events, One-Class Support Vector Machines were trained on a window of time prior to the event, which established a PB-state baseline. The model then evaluated participant's PB-state on the day of the life event and each day that followed, assigning each day a level of deviance relative to the participant's baseline. Resulting response curves were then examined in association with the use of various coping strategies using Bayesian gamma-hurdle regression models. The results from our objectives suggest that physical determinants of health also deviate in response to negative life events and that these deviations can be mitigated through different coping strategies. Taken together, these observations stress the need to examine physical determinants of health alongside psychological determinants when investigating the effects of negative life events.

## 1. Introduction

Ranging from the death of a loved one to injury or illness, negative events are an unavoidable part of life (1). These events are not only overwhelming stressful, they can also have prolonged adverse effects, such as the development of mental health disorders, including depression, anxiety, and Post-traumatic Stress Disorder (PTSD) (2–4). Given their distressing and inevitable nature, a wealth of research has developed around the response to negative life events in an effort to understand how the long-term impact of these events may be mitigated (1, 5).

Traditionally, investigations into the consequences of negative life events have focused on capturing the individual's psychological response (5). To accomplish this, studies have utilized a variety of self-report psychological measures ranging from daily levels of positive and negative affect to yearly reports of life satisfaction (6, 7). While, these efforts have succeeded in quantifying psychological responses to negative life events, the mental effects of negative life events, though important, represent only one dimension of our health. Notably, a person's *physical* health may also be affected by negative life events; however, this dimension has yet to be thoroughly studied.

Investigations into the effects of negative life events on physical health are important for several reasons. First, there is evidence that considerable variance exists in how people respond to negative life events, suggesting that while not everyone will develop prolonged mental health complications, negative consequences of these events may still be experienced in other ways (5). For example, just as these events may lead to increased negative affect or the development of PTSD in some, so too might they lead to poor sleep or diminished physical activity in others. With investigations primarily targeting psychological factors, the latter may go unobserved in large-scale studies. Second, studying physical health may also supplement psychological research, given the well-established connections between physical and mental health, such as increased exercise reducing depressive symptoms and poor sleep associated with increased odds of depression and PTSD (6, 8–13). Including these physical factors may provide additional context to the presence (or absence) of adverse mental health effects following a negative life event (14).

To quantify these physical health factors, traditional self-report surveys have often been utilized; however, such reports are subject to recall bias and inability to capture real-time responses. Instead, recent advancements in wearable technology provide the opportunity to capture attributes of physical health in a continuous and unobtrusive manner, allowing for measures such as resting heart rate, physical activity, and sleep to be captured passively and long term (15–17).

This article serves to leverage this technology to capture attributes of physical health and address this gap in negative life events literature. Our study features a cohort of working professionals located throughout the United States who were observed via Garmin fitness trackers for 1 year and retrospectively recorded any negative life events that occurred while under observation. Utilizing these cohorts' data, we aim to answer two research questions. The first question (RQ1) asks *Does an individual's physiological and behavioral state deviate following a negative life event?* By capturing physiological and behavioral responses to negative life events, we can begin to understand whether attributes of one's physical health can also be perturbed following the event. Once these physical responses to negative life events have been quantified, our secondary aim seeks to assess the relationship between the varying trajectories of these responses and the use of coping strategies. Specifically, the second research question (RQ2) asks *Does the magnitude and duration of deviation associate with how the individual copes with the negative life event?* Coping strategies often depict how people mitigate and solve stressful encounters, and have been organized by (18) into 14 different categories (19, 20). Addressing this question will seek to contextualize individual's physical responses within known factors of psychological responses to negative events, beginning to bridge these two dimensions of health with regard to negative life events.

## 2. Materials and Methods

### 2.1. Study

The data utilized in this article come from the Tesserae study, which recruited 757 participants throughout different companies across the United States, concentrated around four major organizations (21). The study followed participants for 1 year, collecting demographics, psychometrics, fitness tracker data, and life events. Demographics, psychometrics, and life events were collected through surveys given at the beginning and end of the study. Participants' heart rate, physical activity, and sleep were captured through Garmin Vivosmart 3 fitness trackers. To ensure completeness in the data collection, participants were required to wear their Garmin 80% of the time and received monetary compensation if this threshold was met. For a complete detailing of the study, we refer to the reader to (21).

### 2.2. Data

To address the underlying research questions presented in this article, two primary sources of data were required: fitness tracker and survey data. Details for each are provided in this section.

#### 2.2.1. Fitness Tracker Measures

To capture an individual's physiological and behavioral state, we utilized three measures monitored by the Garmin fitness trackers, specifically resting heart rate, physical activity, and sleep. We briefly summarize the importance of each respective measure and detail how they are computed via the Garmin devices.

- **Resting Heart Rate**: Resting heart rate has become a well-established biomarker for cardiovascular health. Following an array of studies, higher RHR has been observed to be independently associated with increased risk of all-cause and cardiovascular mortality (15, 22–26).
  Calculated on a daily basis, Garmin computes RHR as the average of all heart rate readings recorded while the user was asleep, excluding periods where any steps were detected or the readings fell outside reasonable bounds (27).
- **Physical Activity**: An important behavior for reducing all-cause mortality and extending life expectancy, moderate-vigorous physical activity (MVPA), such as brisk walking or running, has been linked to the prevention of many chronic diseases, as well as boosting the immune system and lowering stress levels (16, 28–33).
  Daily minutes of MVPA were defined using Garmin's “Intensity Minutes” measure. Garmin notes these minutes are calculated based on heart rate: comparing the individual's current rate to their average resting heart rate, as well as number of steps taken per minute (34).
- **Sleep duration**: Sleep, or lack thereof, has been repeatedly linked to adverse medical conditions and decreased life expectancy, as such, ensuring 7–9 h of sleep per night is critical to maintaining a person's personal health and wellness (17, 35–37).
  Nightly hours of sleep were computed by Garmin through a combination of the device's heart rate sensor and accelerometer to determine bedtimes and waketimes (38).

In combination, these health measures provide a multidimensional, daily representation of an individual's physiological and behavioral state. For brevity, we further reference this representation as a person's PB-state. To ensure these measures capture different aspects of an individual's PB-state, we conducted a Pearson correlation analysis between each pair of variables within each participant. Across participants, the median (min, max) correlations were 0.02 (−0.13, 0.24) for resting heart rate and sleep duration, 0.06 (−0.09, 0.27) for resting heart rate and active minutes, and −0.006 (−0.12, 0.23) for active minutes and sleep duration. A detailed overview regarding the full distributions of these correlations is provided in Supplementary Figure 1. For the majority of participants, we observe only weak correlations between these measures, with the strongest observed correlation for any one individual being *r* = 0.27. These observations suggest that while these measures may be weakly associated with one another, each offers an independent contribution toward a person's PB-state.

#### 2.2.2. Survey Data

##### 2.2.2.1. Life Events

Following completion of the Tesserae study, a follow-up survey was administered to participants. As part of this survey, participants were asked to detail significant events they had experienced while under observation.

For each event, participants were asked to provide additional details including whether the event was a positive or negative experience (referred to as “valence”), significance of the event, date the event occurred, and their confidence that the date they provided was correct. Valence, significance, and date confidence were each asked on a 7-point Likert scale. For valence, “1” indicated that the event was “Extremely Positive” and “7” indicated that the event was “Extremely Negative.” For significance, “1” indicated “Lowest significance” and “7” indicated “Highest significance.” And for date confidence, “1” indicated “Lowest confidence” and “7” indicated “Highest confidence.” Finally, participants were asked, if willing, to provide a brief description of the event. Such responses included “death of a family member” and “took on a greatly increased work load.”

##### 2.2.2.2. COPE Inventory

Alongside the life events questions, the Brief COPE inventory was included in the follow-up survey. Brief COPE consists of 28 questions to gauge the extent to which the respondent utilizes (if at all) 14 different strategies for coping with adverse events (18, 39). Below, we list the 14 different strategies and provide a brief description for each using excerpts from the author and the specific survey questions when necessary.

**Acceptance** - “...accepts the reality of a stressful situation...” (39)
**Active Coping** - “...the process of taking active steps to try to remove or circumvent the stressor or to ameliorate its effects” (39)
**Behavioral Disengagement** - “...reducing one's effort to deal with the stressor, even giving up the attempt to attain goals with which the stressor is interfering” (39)
**Denial** - “...refusal to believe that the stressor exists or trying to act as though the stressor is not real...” (39)
**Humor** - “...making jokes about it [the stressor]/making fun of the situation...” (18)
**Planning** - “...thinking about how to cope with a stressor. Planning involves coming up with action strategies, thinking about what steps to take and how best to handle the problem” (39)
**Positive Reframing** - “...construing a stressful transaction in positive terms...” (39)
**Religion** - “...the tendency to turn to religion in times of stress” (39)
**Self-Blame** - “...criticizing oneself for responsibility in the situation...” (18)
**Self-Distraction** - a focus on “...doing things to take one's mind off the stressor” (18)
**Substance Use** - “using alcohol or other drugs to feel better/to help me get through it [the stressor]” (18)
**Use of Emotional Support** - “...getting moral support, sympathy or understanding” (39)
**Use of Instrumental Support** - “...seeking advice, assistance, or information.” (39)
**Venting** - “...the tendency to focus on whatever distress or upset one is experiencing and to ventilate those feelings” (39)

### 2.3. Cohort Selection

**Figure 1:**
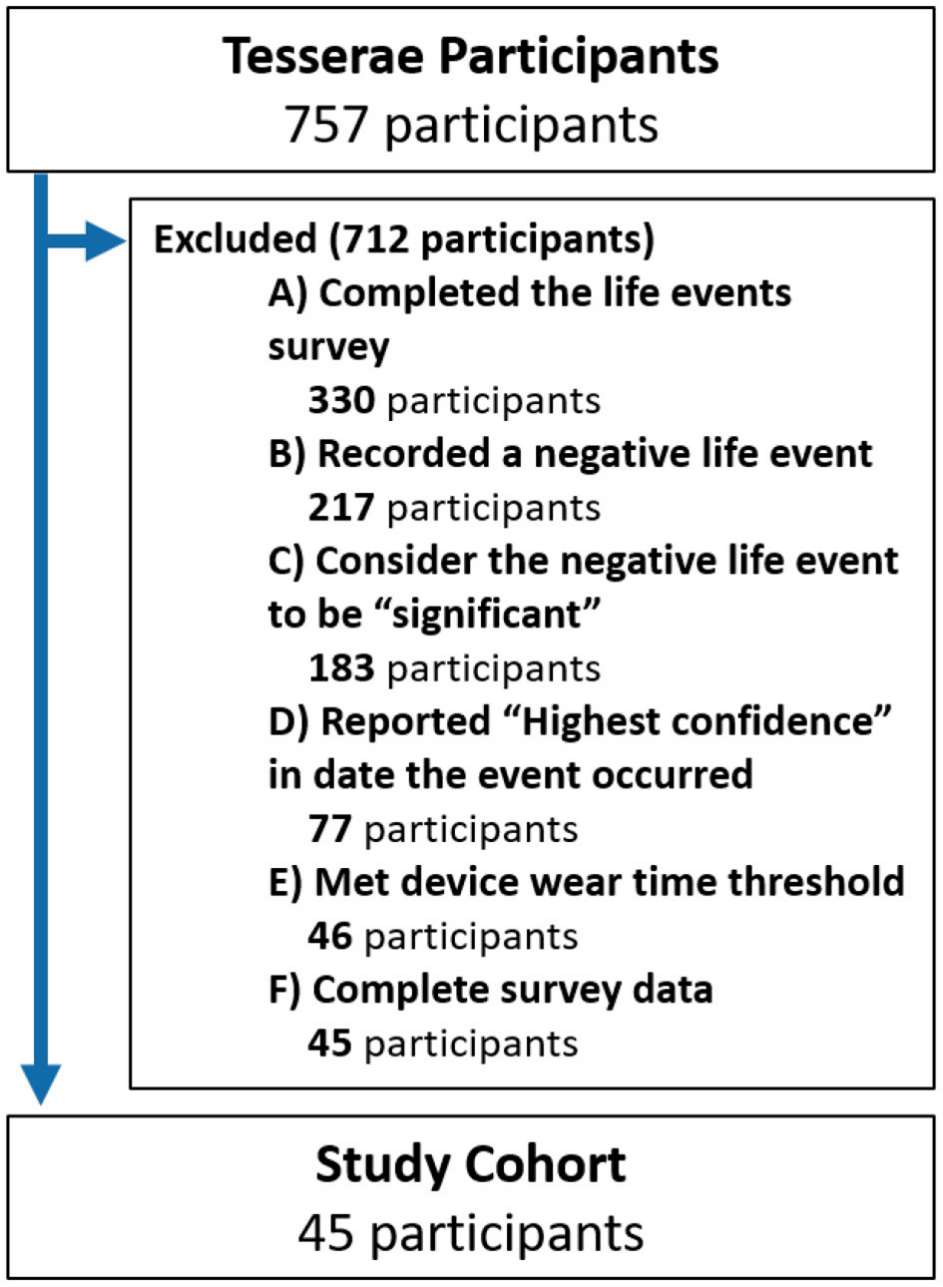
Consort diagram.

Despite enrolling a total of 757 participants, not all were eligible for analysis. To ensure our research questions were appropriately and rigorously addressed, several data filtering steps were required. A full outline of our cohort selection is provided in Figure 1.

The first step in our cohort selection pertains to the life events survey. Among the 757 study participants, a total of 330 participants completed the life events survey during their exit testing. The survey asked participants to report any life events they experienced during the study and describe them by their valence and significance. Additionally, as this survey was asked retrospectively, participants were also required to report their confidence in the accuracy of date they provided for when the event occurred. As the aim of our work was to investigate the effects of major negative life events, several filtering criteria were imposed on these three dimensions. First, only negative life events were considered, resulting in 217 participants with eligible events. To ensure high impact or *significant* life events were studied, only negative events described as having at least “high significance” were included, resulting in 183 participants. Including these criteria ensured that while two participants may have experienced different events, their perception of the valence and significance of the events was comparable. Examples of these life events included marital problems, financial issues, pregnancy complications, and the death of loved ones. Finally, ensuring the life event occurred on the exact date reported was critical, as such, only events marked as having the “Highest confidence” in the date they occurred were considered, resulting in 77 participants with eligible life events.

The next step in our filtering process focused on the amount of time participants wore their fitness trackers. Criteria at this step ensured all individuals were observed via their fitness trackers for an amount of time sufficient to adequately capture their PB-state. This required participants to have worn their device for at least 80% of the days they were observed before and after the negative life event (we elaborate on these “before” and “after” time spans in the next section) and for at least 80% of each day (19 out of 24 h). This 80% threshold was utilized as it has previously been shown to provide reasonable representations of daily of physical activity and sleep (40). Following this step, a total of 31 participants were excluded, resulting in 46 participants.

Lastly, one participant was removed as they did not complete the COPE survey, resulting in a final cohort of 45 participants.

Among this cohort were a total of 26 (57%) females and 19 (42%) males. The age distribution for this cohort was (Min: 23; Q1: 28, Median: 37, Q3: 47, Max: 63).

### 2.4. Analysis

#### 2.4.1. Quantifying Response to Negative Life Events

##### 2.4.1.1. Experiment Setup

To determine whether an individual's PB-state deviated in response to a negative life event (RQ1), it was first necessary to establish each participants “normal” or baseline PB-state prior to the negative life event. This baseline would allow us to assess the degree of deviation from this state for each respective day following the negative life event.

To make these comparisons, participant's multivariate time series, composed of daily measures of resting heart rate, physical activity, and sleep, were partitioned into two windows relative to when the negative life event occurred. First, a 60-day pre-event time window encompassed the days leading up to the negative life event, providing the time-span to establish a baseline. Second, a 15 day post-event window provided the days to be compared against the baseline to assess deviations. Despite the *post-event* window label, we clarify that the post-event window includes the day of the negative life event. Utilizing these windows also ensured that all participants were compared in the same manner.

Finally, we note that while participants could mark multiple significant negative life events throughout their time in the study, we focused only on the first significant negative life event they recorded. This ensured a significant negative life event was not present in the data used to establish a participant's baseline PB-state.

##### 2.4.1.2. Data Pre-processing

Before making comparisons between the pre- and post-event windows, several data pre-processing steps were necessary. Referring back to our cohort selection, all participants were required to have worn their fitness tracker for at least 80% of the days in the pre- and post-event windows, respectively. This threshold ensured an accurate PB-state baseline was established and a sufficient number of days were available to capture the person's response to the negative life event. Within both these windows, any missing daily measures of resting heart rate, physical activity, or sleep duration were imputed using linear interpolation.

Our next step was to address the seasonality and stationarity of the time series data; this would ensure days considered “deviations” were not simply due to the presence of trends in the data or cyclic changes manifesting from the shift between weekdays and weekends. Seasonality was addressed by computing the mean value for each univariate time series for each day of the week and subtracting the mean value from each respective day (41). The deseasonalized time series were then tested for stationarity using an Augmented Dickey-Fuller test on each univariate time series (42). Results of these tests indicated all time series were stationary (α = 0.05).

Lastly, each participant's time series was normalized using z-normalization to remove the magnitude of the different variables, ensuring one variable could not drive the presence of deviations by shifting in greater absolute values. These data pre-processing steps resulted in multivariate time series spanning 75 days, composed of an individual's normalized resting heart rate, total MVPA minutes, and nightly sleep duration, providing a daily representation of an individual's PB-state.

##### 2.4.1.3. Model Specification

To establish a baseline PB-state and measure an individual's response to a negative life event, we framed this task as a novelty detection problem. The goal of novelty detection is to determine, based on a set of training data, whether a new observation is an inlier or outlier (aka a *novelty*) (43). This strategy appropriately addressed our research question, considering a separate model could be trained for each participant using the PB-state data in their pre-event window. Using this learned representation as a participant's baseline PB-state, the model could then parse each day in the participant's post-event window, treating the days as new observations and determining whether each day's PB-state was an inlier or outlier, relative to that participant's learned baseline. This sequence of estimates from the model could then represent the participant's response curve, with days deemed “inliers” representing days where the PB-state was similar to baseline and days deemed “outliers” representing days where the PB-state deviated from the baseline.

The specific model selected for this task was a One-Class Support Vector Machine (OCSVM). An OCSVM was ideal for modeling this phenomenon for several reasons. First, for its ability to account for the multivariate nature of the data by representing a person's daily resting heart rate, physical activity, and sleep as points in a three-dimensional space. Second, utilizing a Radial Basis Function (RBF) kernel allowed for non-linear relationships between the data streams to be captured within the pre-event data (44). Finally, the model provided a singular metric of distance from the distribution of training data using the learned decision boundary, effectively capturing the *degree* to which each post-event day was considered an inlier or outlier (45). Days which fell within the bounds of the decision function (inliers) were represented by positive values, and days which fell outside the bounds of the decision function (outliers) were represented by negative values; the magnitude of the values indicated the degree to which a single day was an inlier or outlier. Classifying each day across the post-event window resulted in a univariate sequence of values, representing the negative life event response curve. Daily values in this sequence represented the degree to which the individual conformed to or deviated from their baseline PB-state.

OCSVMs were implemented using scikit-learn (v0.21.3) (46, 47). All OCSVM parameters were held constant across the separate models trained for each participant. By holding these parameters constant, we ensured the detection of novel (or outlying) days did not result from variance in model parameters between participants and arose strictly from the distribution of their PB-state baseline. The parameter ν, which designates the upper bound on the fraction of training errors, was set to an arbitrarily small value (0.01) to ensure the OCSVMs trained on all pre-event data when learning the PB-state baseline. Parameters other than ν utilized the default settings in scikit-learn, which can be found in the scikit-learn documentation (46, 47).

##### 2.4.1.4. Internal Validation

Finally, to ensure any observed deviations in PB-state were not due to random chance, an internal validation study was conducted. The objective of this study was to compare response curves generated following the day of a negative life event to curves generated following a day with no event, with the latter group of curves acting as a control group. We could reasonably assume that if the curves from both groups are similar, than the deviations would likely have occurred by chance. However, if the deviations in the “life events” group were stronger than the deviations in the “no events” group, this would provide evidence that deviations following a negative life event may be associated with the event.

This experiment was conducted as follows: for each individual, we began with their full time series; representing their entire time in the study. We then removed the 75-day window (60 days pre-event, 15 days post-event), which was utilized for investigating their response to a negative life event. A sliding window of 75 days then swept across their remaining time series, sliding on a day-by-day basis, capturing all valid, contiguous 75 day time blocks. A time block was considered valid if (1) it met the same 80% wear time threshold used in the initial analysis and (2) no life event was present in the block.

Having collected valid time blocks for each participant, these blocks were then bootstrapped (sampled with replacement) 10,000 times, with an OCSVM model training on the first 60 days of each block and classifying the last 15 days (48). This resulted in 10,000 *no event* response curves for each individual. From these, the median response curve was then computed for each participant, effectively estimating a generalized median response to *no event* for each participant. These *no event* response curves were then compared to the response curves, which resulted from negative life events with two tests.

To determine whether a significant difference existed between the negative life event and *no event* response curves, two comparisons were made in accordance with our two response characteristics of interest: *immediate impact* and *short-term trajectory*, detailed in the next section. The first test is aligned with *immediate impact*, assessing deviations on the first day of the response. This was done using the non-parametric Wilcoxon signed-rank test, comparing the paired distributions of event-day deviations resulting from the life event to event-day responses resulting from *no event*. The second test was aligned with *short-term trajectory* and was performed on the sum of deviations within the response curve to assess the cumulative deviation in response to an event. Again, a Wilcoxon test was performed assessing the paired distribution of cumulative deviation in response to a negative life event to the cumulative deviation in response to *no event*.

#### 2.4.2. Response Characteristics

Moving to RQ2, we sought to investigate how the utilization of various coping strategies may be associated with these negative life event response curves. This question was partitioned into two sub-questions based on the two characteristics of the response curve that were of interest. The first characteristic being the degree of deviation on day 1 of the curve or the *immediate impact* on PB-state in response to the negative life event. The second, being the overall shape of the curve or *short-term trajectory* spanning the 2 weeks that followed the life event. This would answer questions such as whether the deviations persisted? or did they quickly return to baseline?

Using each of these characteristics as an outcome, associations between them and each coping strategy were modeled using a series of Bayesian regressions, while adjusting for several potential confounding variables. We note a Bayesian approach was taken for this analysis as previous studies have shown Bayesian methods to yield more accurate estimates among small data (49–52). Bayesian regressions were implemented using the R package BRMs (53) and statistics were derived from these models using the R package BayesTestR (54).

**Immediate Impact** - The first characteristic of the response curve we investigated was immediate impact, or response on the day of the negative life event. This characteristic was chosen as previous works examining response to negative life events observed the most significant changes to occur immediately following the event (6, 7, 55). Immediate impact was measured by evaluating the degree of deviation a participant experienced on the day the negative life event occurred. As the OCSVMs provided the degree to which a day was an inlier as well as an outlier, we truncated the degree of all inliers to 0. Given this study was specifically focused on deviation, knowing the degree to which a person's PB-state was an *inlier* was unnecessary, considering the model had already determined an inlier to be representative of a person's normal PB-state. To more easily model these outliers, we switched their original negative sign to positive. This resulted in a right-skewed distribution where absolute zero represented normal behavior and positive values represented the degree of deviation. To appropriately model this mix of absolute zeros and strictly positive values, we utilized a Gamma hurdle family for our Bayesian models (53). A series of regression tests were then performed to investigate each coping strategy covered in the COPE inventory. Coping strategies were analyzed separately to allow the models parameter space to better fit recommended parameter-to-sample ratios and prevent the correlations among these coping strategies from producing biased estimates (56, 57). Each regression adjusted for demographic traits: gender and age, as well as the participant's perceived valence and significance of the event. For each regression, we utilized uninformative flat priors and a total of 100,000 posterior samples were drawn, ensuring the necessary 10,000 effective samples recommended for stable credible intervals (58).
**Short-term trajectory**: The second characteristic of the response curves we investigated was the short-term trajectory, allowing us to assess whether deviations persisted across the 2 weeks following the negative life event. Once again, all inliers were truncated at zero, but this time, outliers retained their negative sign. To capture common response trajectories, each individual's response curve was first smoothed using lowess, after which *K*-shape clustering was performed on the smoothed response curves (59). This resulted in an optimal *K* of two, based on silhouette score. After identifying these two clusters, associations between response curves and coping strategies were tested using Bayesian logistic regression. We utilized the same procedure for adjustment variables, sampling iterations, and credible intervals as we did for the *immediate impact* characteristic.

#### 2.4.3. Common Deviations

Having addressed the two research questions posed in this article, we concluded with a *post hoc* analysis to determine whether common deviations existed within the PB-state response to a negative life event. Utilizing the clustering outcomes from the previous section, we investigated whether a significant group-level increase or decrease in resting heart rate, physical activity, or sleep duration manifested following the negative life event.

Median values were computed for each individual within their pre-event and post-event windows for each of these three measures. Utilizing paired-distribution Wilcoxon tests, we tested whether a significant difference existed in each variable between the median pre-event values and median post-event values. Tests were performed on each cluster of participants separately.

Before concluding the Methods section of this article, we briefly comment on statistical significance. Given the recent movements toward reevaluating statistical significance as a scale rather than binary operation, we provide exact values for all data examined and allow the reader to decide their own interpretation of “significance” (60).

## 3. Results

### 3.1. Response to Negative Life Events

**Figure 2:**
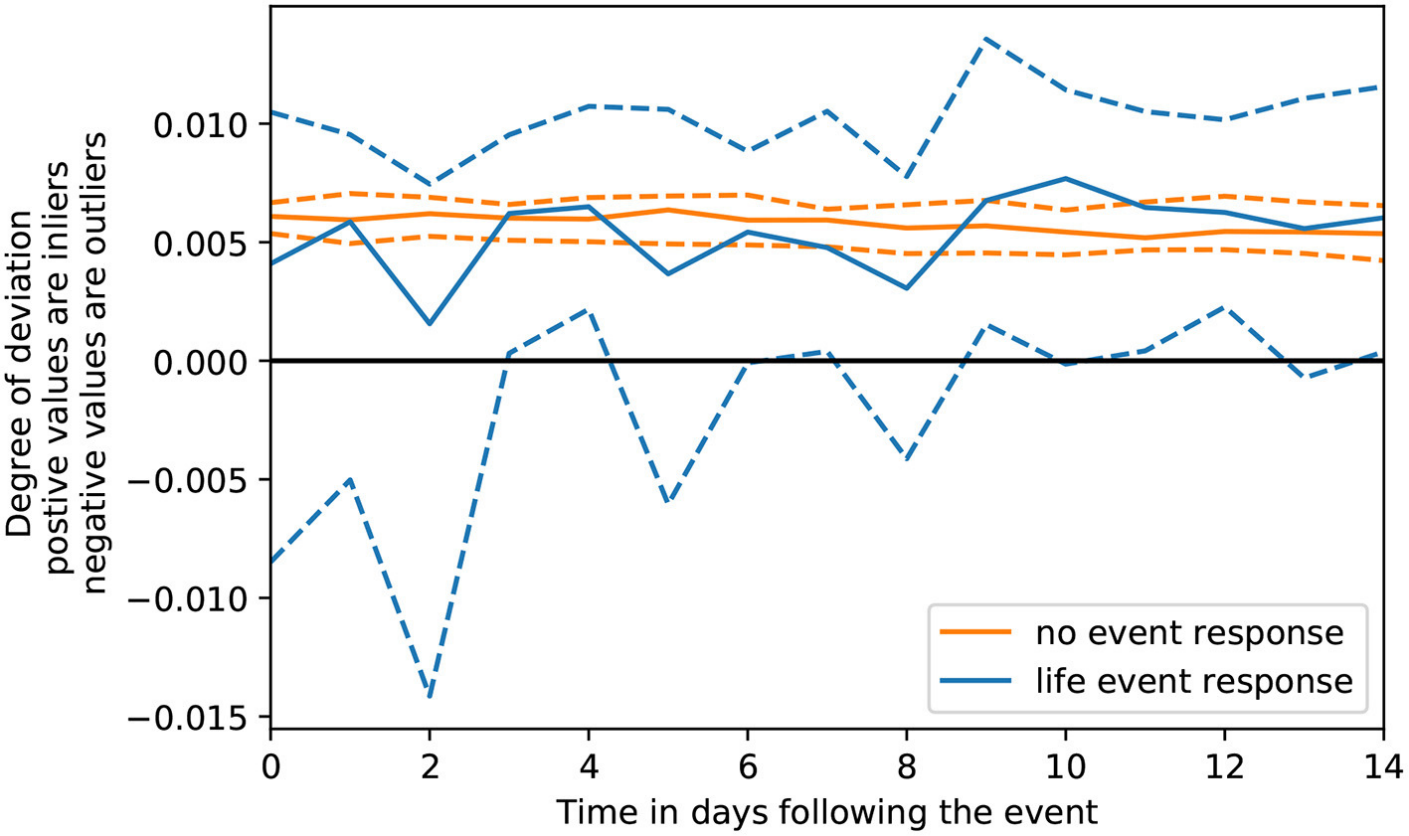
Distribution across time of negative life event response curves compared to no event response curves.

To address RQ1, OCSVMs were trained on a participant's pre-event window to learn their baseline PB-state. These models then parsed the participant's post-event window, estimating for each day, whether the observed PB-state was an inlier or outlier, respective to the learned baseline. This sequence of daily estimates represented the participant's negative life event response curve. Daily values of these response curves represented the degree to which that day's PB-state conformed to (represented by positive values) or deviated from (represented by negative values) the learned baseline, with larger positive values indicating stronger conformity and smaller negative values indicating stronger deviation. An aggregation of these sequences is provided in Figure 2. The solid blue line represents the median estimate across all participants, while the blue dashed lines represent the 25th and 75th percentiles (the yellow lines will be covered in the next paragraph). We observe that the median response remains positive across the post-event window, while the lower quartile is negative and slowly becomes positive over the post-event window.

With respect to the validation study, the yellow lines in Figure 2 represent an aggregation of response curves in which no negative life event was present. Similar to the blue lines, the solid yellow line represents the median and dashed yellow lines represent the 25th and 75th percentiles. Using these two groups of curves, we can reasonably assume that if the same degree of deviation was found in both the yellow and blue lines, then the deviations in the blue lines may be due to random chance, as response curves generated when no negative life event was present, would produce the same degree of deviation as curves generated when an event was present.

To determine whether these response curves were significantly different, we performed two statistical tests in line with our two characteristics of interest. The first test assessed immediate impact of the response. From our Wilcoxon signed-rank test, we observed that deviations on the first day of the curve were significantly stronger in response to the negative life event than deviations on the first day of the curve in response to *no event* (*P* = 0.014). The second test assessed short-term trajectory by comparing the sum of deviations across the response curve. Again, using a Wilcoxon test, we observed that the sum of deviations across the negative life event response curves were significantly stronger than the sum of deviations across the *no event* response curves (*P* < 0.001). Overall, the results from this internal validation analysis provide evidence that the deviations observed following the negative life event were not due to random chance.

### 3.2. Response Characteristics

Moving to RQ2, we modeled the association between two characteristics of the negative life event responses: immediate impact and short-term trajectory, with different coping strategies through a series of Bayesian regressions. For both response characteristics, we provide the median effect size and 90% credible interval of each COPE measure. For convenience, this information is also displayed as a coefficient plot for each response characteristic. Additionally, we provide the probability of direction for each measure; an index of effect existence based on the posterior distribution which ranges from 50 to 100%. The index represents the certainty with which an effect goes in a given direction, such as positive or negative (54, 61). Strongly correlated with *P*-values, a probability of direction of 95% is roughly equivalent to a *P*-value of 0.1.

#### 3.2.1. Immediate Impact

**Figure 3:**
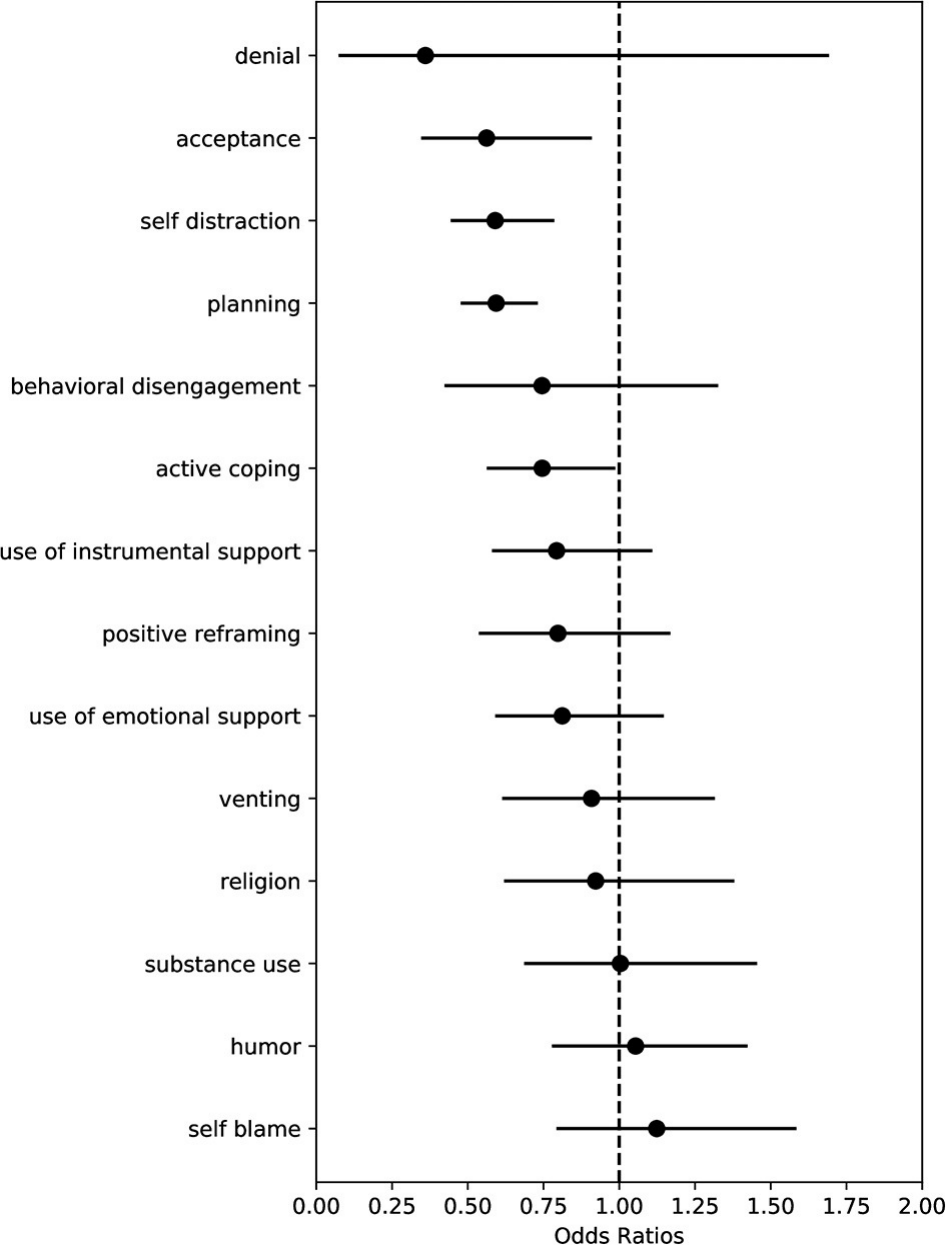
Coefficients plot of COPE measures associated with event day deviation. Points indicate median effect size and black horizontal lines indicate the range of the 90% credible interval.

Observations for associations between coping strategies and immediate impact are detailed in Figure 3, with numerical values and probability of direction provided in Table 1. Having utilized a gamma family for our regressions, values above 1 represent the multiplicative increase in deviation on the day of the event, while values below 1 represent the multiplicative decrease in deviation on the day of the event.

**Table 1:** Coefficients and probability of direction associated with event day deviation.

**Coping strategy**	**Coefficient (90% CI)**	**Probability of direction**
Acceptance	0.56 (0.35, 0.90)	0.972
Active coping	0.75 (0.57, 0.98)	0.955
Behavioral disengagement	0.75 (0.43, 1.32)	0.811
Denial	0.36 (0.08, 1.69)	0.868
Humor	1.05 (0.78, 1.42)	0.623
Planning	0.59 (0.48, 0.73)	0.999
Positive reframing	0.80 (0.54, 1.16)	0.847
Religion	0.92 (0.63, 1.37)	0.638
Self-blame	1.12 (0.80, 1.58)	0.722
Self-distraction	0.59 (0.45, 0.78)	0.996
Substance use	1.00 (0.69, 1.45)	0.507
Use of emotional support	0.81 (0.60, 1.14)	0.846
Use of instrumental support	0.79 (0.59, 1.10)	0.880
Venting	0.91 (0.62, 1.31)	0.674

Utilizing the Brief COPE inventory, we observed several coping strategies significantly associated with less deviation. Specifically, we observe that *acceptance* (OR 0.56; 90% CI: 0.35, 0.90), *self-distraction* (OR 0.59; 90% CI: 0.45, 0.78), *planning* (OR 0.59; 90% CI: 0.48, 0.73), and *active coping* (OR 0.75; 90% CI: 0.57, 0.98) were significantly associated with less deviation regarding the immediate impact of the negative life event.

#### 3.2.2. Short-Term Trajectory

**Figure 4:**
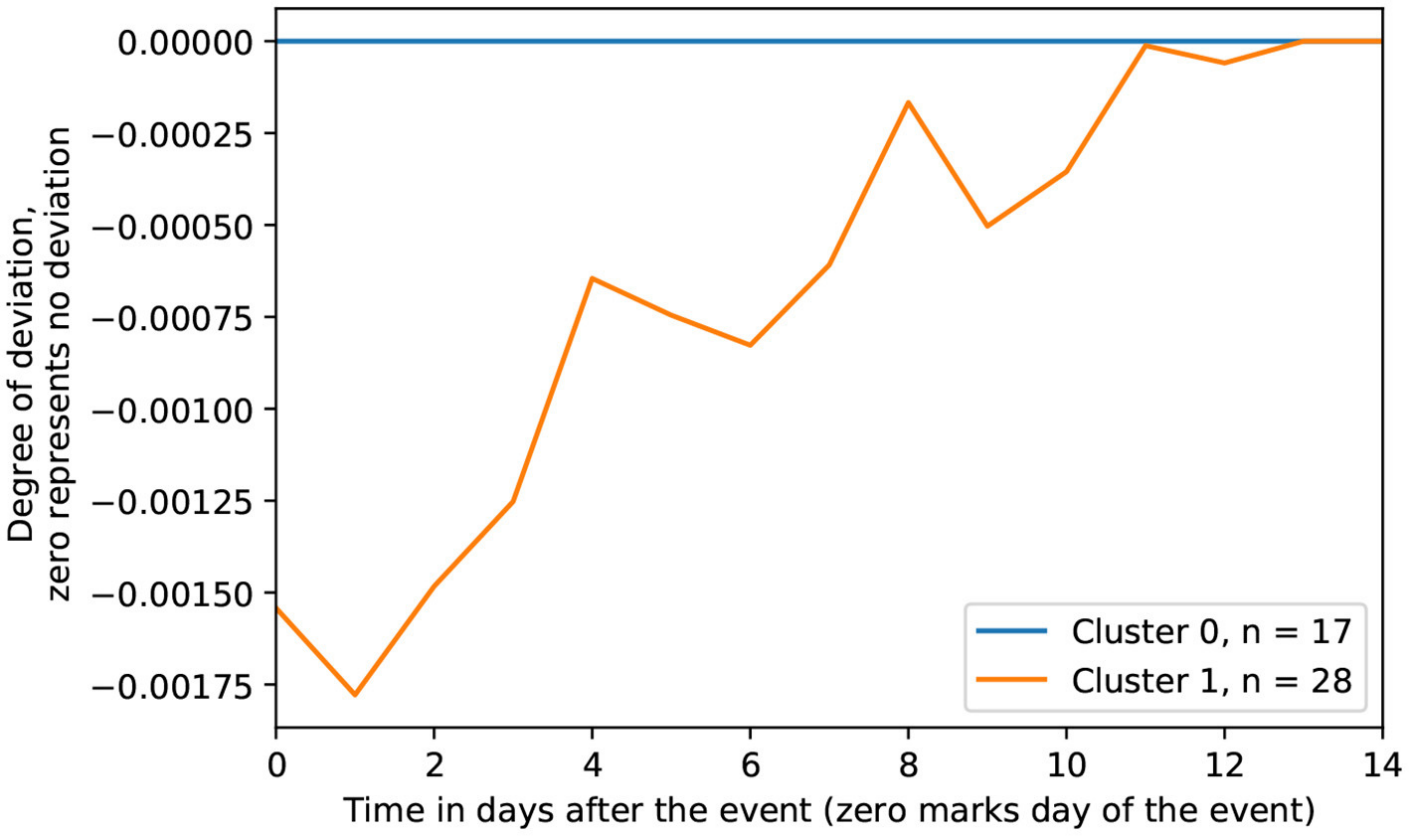
Clustered response trajectories.

To capture common short-term trajectories among the response curves, K-shape clustering was utilized, producing a optimal partition of two clusters. Figure 4 visualizes the median degree of deviation for each clustering of response curves across the post-event window. Cluster 0 (*n* = 17) depicts minimal deviation in response to the negative life event, whereas Cluster 1 (*n* = 28) shows an immediate deviation on the day of the negative life event and gradual return to baseline PB-state across the following 2 weeks.

**Figure 5:**
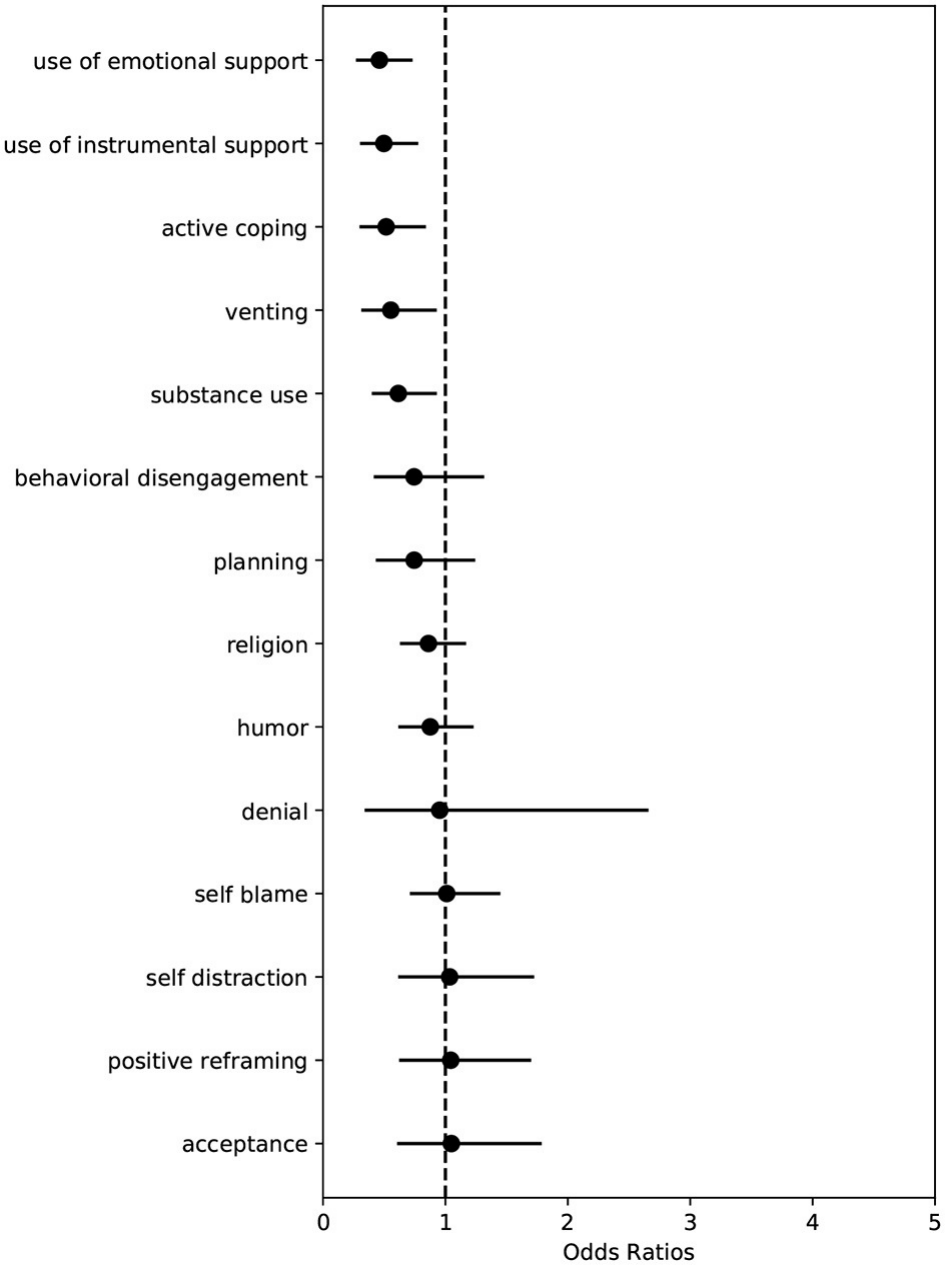
Coefficients plot of COPE measures associated with response cluster. Points indicate median effect size and black horizontal lines indicate the range of the 90% credible interval.

Associations between coping strategies and clusters are provided in Table 2. A visual representation of the effect sizes can be found in Figure 5. In both cases, values are represented as odds ratios, with values above 1 indicating a higher likelihood of belonging to Cluster 1, while values below 1 indicate a lower likelihood of belonging to Cluster 1.

**Table 2:** Coefficients and probability of direction associated with response cluster.

**Coping strategy**	**Coefficient (90% CI)**	**Probability of direction**
Acceptance	1.05 (0.62, 1.77)	0.559
Active coping	0.52 (0.31, 0.83)	0.993
Behavioral disengagement	0.74 (0.43, 1.30)	0.813
Denial	0.95 (0.35, 2.65)	0.532
Humor	0.88 (0.63, 1.22)	0.748
Planning	0.74 (0.44, 1.23)	0.840
Positive reframing	1.04 (0.64, 1.69)	0.557
Religion	0.86 (0.64, 1.16)	0.802
Self-blame	1.01 (0.72, 1.44)	0.521
Self-distraction	1.03 (0.63, 1.71)	0.544
Substance use	0.62 (0.41, 0.92)	0.984
Use of emotional support	0.46 (0.28, 0.72)	0.999
Use of instrumental support	0.50 (0.32, 0.76)	0.998
Venting	0.55 (0.33, 0.92)	0.980

We observe several coping strategies significantly associated with the cluster in which minimal deviation was observed. Specifically, we observe that *active coping* (OR 0.52; 90% CI: 0.31, 0.83), *substance use* (OR 0.62; 90% CI: 0.41, 0.92), *use of emotional support* (OR 0.46; 90% CI: 0.28, 0.72), *use of instrumental support* (OR 0.50; 90% CI: 0.32, 0.76), and *venting* (OR 0.55; 90% CI: 0.33, 0.92) were significantly associated with increased odds of belonging to Cluster 0. No coping strategies were observed to be significantly associated with increased odds of belonging to Cluster 1.

### 3.3. Common Deviations

Finally, our *post hoc* analysis focused on the three underlying components of a person's PB-state to assess whether common deviations existed within the response curves. Referring to Table 3, for Cluster 0, we observed no significant changes in resting heart rate (*P* = 0.77), physical activity (*P* = 0.40), or sleep (*P* = 0.52). For Cluster 1, however, a significant increase in resting heart rate was observed (*P* = 0.06) with an average increase of 0.66 bpm in the post-event window relative to the pre-event window. A significant decrease in physical activity was also observed (*P* = 0.06) with an average decrease of 4 active minutes per day. No significant changes in sleep duration (*P* = 0.81) were observed for Cluster 1.

**Table 3:** Average change in metric between pre- and post-event stratified by cluster.

	**Cluster 0**		**Cluster 1**	
	**Mean change**	***P*-value**	**Mean change**	***P*-value**
Resting heart rate	−0.20 bpm	0.77	+0.66 bpm	0.06
Daily physical activity	+1.05 min	0.40	−4.10 min	0.06
Nightly sleep duration	0.10 h	0.52	+0.02 h	0.81

## 4. Discussion

### 4.1. Principal Findings

In this article, we aimed to answer two research questions: RQ1: *Does an individual's physiological and behavioral state deviate following a negative life event?* RQ2: *Does the magnitude and duration of deviation associate with how the individual copes with the negative life event?*

To address RQ1, we observed two distinct groups of participants, the first group experienced minimal to no deviations following a negative life event, while the second group experienced immediate deviations, with deviations gradually diminishing over the following 2 weeks. These observations support our hypothesis that, in addition to psychological changes, physiological and behavioral changes can also manifest in response to negative life events. Such variations in response manifestation suggest that multiple data modalities may be necessary for detecting if and how a person may be impacted by a negative life event. Moreover, studies that limit these modalities, such as considering only a single psychological measure, may fail to identify individuals affected by the event, but who do not react in a way noticeable by that measure, potentially underestimating the incidence of events. As such, the physical attributes measured by fitness trackers may be useful in capturing the true incidence of subjects affected within a heterogeneous population.

An RQ1 *post hoc* analysis on these physiological and behavioral changes was conducted: breaking down the three measures which represented a person's PB-state: resting heart rate, physical activity, and sleep. Among those who experienced deviations in their PB-state, two common changes were observed: increases in resting heart rate and decreases in physical activity. The increases in resting heart rate may be reflective of concurrent psychological changes as stress and depression have shown to elevate heart rate (62, 63). Regarding physical activity, while the reduction was minimal, this temporary adoption of more sedentary behaviors may be detrimental, as physical activity has been associated with better mental health (8). These findings suggest that overall, the physiological and behavioral changes adopted following negative life events may have a direct impact on one's physical health.

Moving to RQ2, we discuss the association between PB-state deviations and the psychological mechanisms a person may use to cope with negative life events. Overall, we observed that the utilization of coping strategies was generally associated with less deviation in a person's immediate response and short-term trajectory. In particular, we observed “active coping” was significantly associated with less deviation in *both* characteristics. Active coping is defined within the COPE inventory as the extent to which “[the individual has] been concentrating [their] efforts on doing something about the situation [they're] in” and “taking action to try to make the situation better” (18). In other words, the willingness of an individual to proactively engage with and address a problem directly is strongly associated with minimized PB-state deviation. This may suggest that taking steps to remove/alleviate the trauma incurred from a negative life event, through direct mitigation or solutions, concurrently inhibits or diminishes any physiological or behavioral changes.

Taken together, these observations stress the need to examine physical determinants of health alongside psychological determinants when investigating the effects of negative life events. Building upon these findings, future studies hold the opportunity to measure physical and psychological reactions in tandem, leading to a stronger understanding of the connections between physical and psychological responses, providing context for each. Ultimately, by leveraging these holistic views of a person's response to negative life events, we can move closer toward mitigating their adverse developments.

### 4.2. Comparison With Previous Work

Early investigations into the effects of negative life events focused primarily on their psychological consequences (64, 65). Finding associations with the development of anxiety, depression, and PTSD, these works motivated future studies to focus on *why* or *how* these mental health disorders may develop following such events (66–68). Our study builds upon these previous works by exploring a less established component of the negative life events literature: changes in physical health, and by relating these changes to coping strategies, explores how these changes associate with the better understood components of mental health.

Several previous works have begun to investigate the effects of negative life events on different attributes of physical health. These studies have found that levels of physical health can moderate the negative effects of life events, while negative life events can also lead to changes in physical activity and sleep (69–72). Our study builds on these investigations through the utilization of fitness trackers to provide objective and immediate responses to these negative life events and utilizes multiple attributes of physical health simultaneously.

Previous works have also utilized fitness trackers to measure event response, primarily, for stress detection (73–76). Such studies have achieved promising performance using continuous measures of heart rate, skin conductance, and skin temperature. Further, studies have revealed variation in individual's physiological responses, such as more blunted physiological responses to stress (77). While these works are similar to ours in that they measure the physiological effects of negative stimuli, the distinction is made on the type of stimuli being studied. Stress is a constantly applied stimuli where physiological changes occur while it is being applied, for example, a person may be stressed for an hour and may experience an elevated heart rate for that hour. Negative life events, however, are a single stimuli in which an event transpires. Our work not only captures the physiological and behavioral changes that may arise while the event is occurring, it also captures the lasting effects to assess whether changes persist after the event has occurred.

### 4.3. Limitations

The analysis presented in this manuscript is based on a small sample of participants, limiting the generalizability of these findings. As such, the presented results should be considered only as exploratory, requiring follow-up studies with larger sample sizes across various cohorts to validate these findings.

Further, our window for observing deviations from a person's normal PB-state was limited to the 2 weeks following the negative life event. While, we observed gradually diminishing deviations over this period, future studies would benefit from a longer observation period in general, perhaps measuring the years leading up to and following negative life events for the assessment of long-term changes.

Despite pre-event periods containing no significant negative life events, events originally filtered from our analysis may still have been present in these baseline periods. To ensure events occurring in the baseline period did not bias our results, a supplementary analysis was performed showing minimal association between the presence of an event in a person's baseline period and whether they experienced PB-state deviations in response to their negative life event. A complete detailing of this analysis is available in Supplementary Analysis 1.

Finally, it is important to note the limitations of the devices used in this study. Comparing the devices to an ECG, high levels of accuracy for HR monitoring have been observed, especially when the user is at rest (78). For physical activity and sleep, however, Garmin tended to overestimate daily MVPA and total sleep time (79, 80). While, validation studies across larger and more diverse cohorts are needed, current recommendations suggest these devices provide acceptable levels of accuracy, but should be utilized with caution (78–80).

## Data Availability Statement

The datasets presented in this study can be found in online repositories. The names of the repository/repositories and accession number(s) can be found below: http://tesserae.nd.edu/data-sharing/.

## Ethics Statement

The studies involving human participants were reviewed and approved by University of Notre Dame Institutional Review Board and through reliance agreements at participating research universities. The patients/participants provided their written informed consent to participate in this study.

## Author Contributions

LF, KF, and NC designed the study. LF, KF, and SL performed the analysis. LF, KF, SM, SD'M, and NC performed the interpretation of the data. LF, KF, SD'M, and NC wrote and reviewed the manuscript. All authors approved the manuscript for publication.

## Conflict of Interest

The authors declare that the research was conducted in the absence of any commercial or financial relationships that could be construed as a potential conflict of interest.
