# Supplementary material for: Examining Response to Negative Life Events Through Fitness Tracker Data
Source: Front Digit Health. 2021 May 21;3:659088. doi: 10.3389/fdgth.2021.659088 (PMC8521839; doi:10.3389/fdgth.2021.659088)
Supplement: Supplementary file 1 [file Data_Sheet_1.docx]

**Supplementary Figure 1**


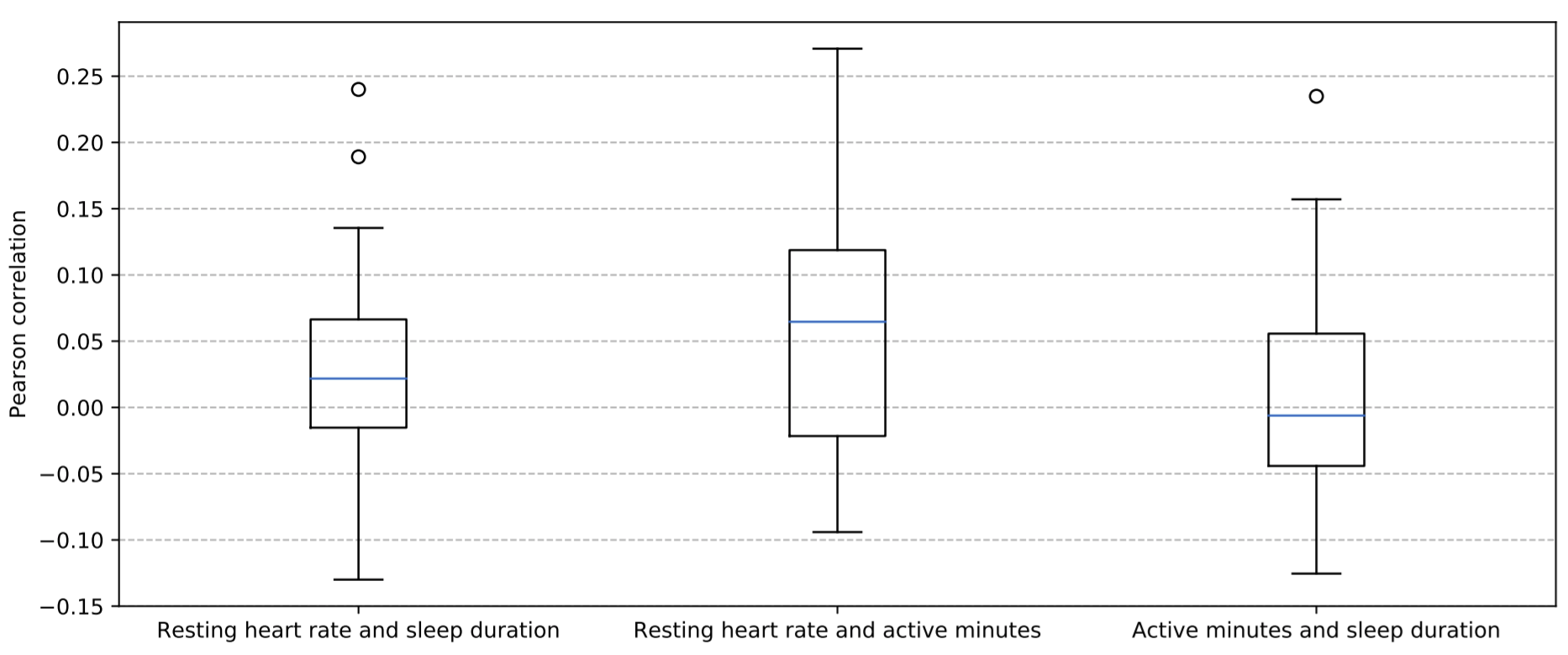


Distribution of within-person correlations between variables for the studied cohort (n = 45).

**Supplementary Analysis 1**

Despite pre-event periods containing no significant negative life events, events originally filtered from our analysis may still have been present in these baseline periods. To ensure events occurring in the baseline period did not bias our results, we performed an additional analysis to assess the relationship between the presence of an event in a person’s baseline period and cluster assignment.

Among the 45 participants studied, a total of 17 participants experienced an event occurring in their baseline period. To assess whether these events may have biased deviations, we conducted a Fisher’s Exact test, comparing cluster assignment with the presence of an event occurring in one’s baseline period. We observed minimal association between presence of a life event and cluster assignment (P = 0.18), with the corresponding contingency table provided in Table S1.

**Table S1**. Contingency table comparing cluster assignment with the presence of an event occurring in a person’s baseline period.

|  | Cluster 0 | Cluster 1 |
| --- | --- | --- |
| Event in baseline | 7 | 6 |
| No event in baseline | 10 | 22 |
| Total | 17 | 28 |
